# Immune Responses of Mango Callus Infected by *Agrobacterium tumefaciens* Inhibited Transformation

**DOI:** 10.3390/ijms26115006

**Published:** 2025-05-22

**Authors:** Haiyan Shu, Zilhas Ahmed Jewel, Omor Faruk, Luqiong He, Qing Wei, Rulin Zhan, Shenghe Chang

**Affiliations:** 1 Tropical Crops Genetics Resources Institute, Chinese Academy of Tropical Agricultural Sciences, Haikou 572025, China; shuhy@catas.cn (H.S.); qingwei_321@163.com (Q.W.); 2 Sanya Research Institute, Chinese Academy of Tropical Agricultural Sciences, Sanya 572025, China; 3 National Key Laboratory for Tropical Crop Breeding, Sanya 572024, China; 4 Key Laboratory of Crop Gene Resources and Germplasm Enhancement in Southern China, Ministry of Agriculture and Rual Affairs, Haikou 571101, China; 5 Key Laboratory of Tropical Crops Germplasm Resources Genetic Improvement and Innovation of Hainan Province, Haikou 571101, China; 6 Faculty of Agriculture, Gopalganj Science and Technology University, Gopalganj 8100, Bangladesh; zilhas@gstu.edu.bd; 7 Department of Biotechnology and Genetic Engineering, Gopalganj Science and Technology University, Gopalganj 8100, Bangladesh; omor.faruk.bsmrstu@gmail.com; 8 College of Tropical Crops, Yunan Agricultural University, Puer 665099, China; hluqiong@163.com

**Keywords:** browning, gallic acid, infection efficiency, reactive oxygen species (ROS), type III secretion system gene cluster (T3SS)

## Abstract

Mango is a vital fruit crop in tropical and subtropical regions, yet pests and diseases cause 30–70% production losses. Developing disease-resistant cultivars through transgenic methods could mitigate these issues. *Agrobacterium*-mediated callus transformation is a common genetic engineering approach, but successful transgenic mango plants from callus remain unreported due to severe browning and necrosis post-infection. We hypothesized that *Agrobacterium*-induced immune responses trigger callus death, hindering transformation. To improve efficiency, we engineered an *Agrobacterium* strain carrying the type III secretion system (T3SS) and effector gene *AvrPto*. Compared to controls, infected calluses exhibited elevated reactive oxygen species (ROS), along with up-regulated ROS-related, gallic acid biosynthesis, and defense genes. Calluses infected with T3SS-*AvrPto*-harboring *Agrobacterium* showed delayed browning and necrosis versus those infected with the empty vector (NV). The transformation rate with *Agrobacterium* (T3SS-*AvrPto*-EGFP) reached 1.6%, while *Agrobacterium* (NV-EGFP) failed entirely. These findings demonstrate that T3SS and *AvrPto* enhance mango transformation efficiency, offering a promising strategy for breeding multi-resistant varieties.

## 1. Introduction

Mango (*Mangifera indica* L.) is the world’s second-largest tropical fruit in yield annually (2023 data from the Office of Tropical Crops in Southern Asia, Ministry of Agriculture, China). The global mango planting area exceeds 10 million hectares annually (data from the Food and Agriculture Organization of the United Nations). India is the largest mango producer, with a planting area of 2.625 million hectares. In 2023, China’s mango planting area was 386,200 hectares, and the mango output value in 2021 was CNY 21.14 billion. Other major mango-producing countries include Thailand (204,000 hectares), Indonesia (266,700 hectares), Pakistan (213,500 hectares), Mexico (215,600 hectares), Brazil (98,200 hectares), Malawi (69,900 hectares), Bangladesh (139,800 hectares), Egypt (148,300 hectares), and so on.

During mango planting, the losses caused by pests and diseases account for 30–70% of mango production generally [1,2]. Mango anthracnose disease (MAD) can cause up to 100% yield loss in unmanaged orchards [1]. In India, the disease leads to an estimated yield loss of about 39% [1]. In Indonesia, the yield loss due to MAD is reported to be 50.28% [1]. In China, MAD causes an annual yield loss of 30–60% in mango production [1]. The avoidable yield loss caused by mango pests is about 61.51% [2]. Among them, mango hoppers and thrips are the main pests [2]. Cultivating new varieties with various resistances is the basic way to resolve these problems. The conventional method for generating new mango varieties is cross-breeding. A complete cycle of cross-breeding for mangoes always takes about 10–20 years [3]. Transgene has high breeding efficiency and can solve the problems above in a short time [4,5]. After two-week-old mango seedlings were punctured at the apical meristem, the apical meristem was infected with *Agrobacterium* harboring GFP and GUS genes individually [6]. Some plants were found to have been transformed successfully [6]. Adjei et al. (2023) optimized the conditions of the protoplast isolation and transient expression system for mango [7]. GFP fluorescence was observed in the transformed protoplasts [7]. The target gene was successfully induced into the protoplast [7].

Stem apex meristematic tissue is a highly differentiated tissue. When stem apex meristematic tissues were transformed, some cells were transformed and some failed, which always resulted in a chimera [8,9]. When cells are divided, transformation-failed cells always stand ahead of the competition, and the transformed cells stop division, resulting in foreign genes being lost and the failure of the experiment [8,9]. The experiment that Adjei et al. (2023) did was a transient transformation and did not produce transformed seedlings [7]. Plant embryogenic callus is a loose and crisp tissue. When *Agrobacterium* infected the embryogenic callus, callus cells had a high chance of being infected and transformed. Infecting calluses using *Agrobacterium*-harboring target genes is a conventional method for constructing a transgene protocol [10,11,12]. However, the transgenic-mango plant from callus has not been reported until now. In this research, we found that most mango calluses necrotized and died after *Agrobacterium* infected them. No seedling was differentiated from the surviving callus. Immune response can injure and kill plant cells, resulting in callus browned and necrotized [13]. When infecting plant cells, *Agrobacterium tumefaciens* sends virulence proteins into host cells through the type IV secretion system (T4SS) [14]. Active plant defense relying on innate immune responses was triggered [15]. Some Gram-negative plant pathogenic bacteria, such as *Pseudomonas syringae* pv. tomato, deliver bacterial proteins into eukaryotic hosts through the type III secretion system (T3SS), which can escape being perceived by plant cells and do not induce the plant basal defense [15].

We propose that the browning and necrosis of callus tissue after *Agrobacterium* infection are the main reasons for the failure of mango genetic transformation. The browning and necrosis of mango calluses might be due to immune responses triggered by *Agrobacterium* infection. Constructing an *Agrobacterium*-harboring type III secretion gene cluster (T3SS) and the effector gene *AvrPto* might improve the transformation efficiency of mango callus. To identify these hypotheses, metabolome and transcriptome experiments were performed to find substances and genes that play crucial roles in the immune responses of mango callus against *Agrobacterium* infection in this research. An *Agrobacterium* strain harboring T3SS and its effector gene *AvrPto*, isolated from *Pseudomonas syringae* pv. tomato strain DC3000, was constructed. The mango callus infected by *Agrobacterium* (T3SS-*AvrPto*) showed slower browning and necrosis than those infected by *Agrobacterium* harboring the net vector (NV). The transformation rate of mango callus infected with *Agrobacterium* (T3SS-*AvrPto*-EGFP) was about 1.6%, a promising finding encouraging further exploration. These findings provide hope for the potential application of the transgenic method in mango breeding.

## 2. Results

### 2.1. Browning and Necrosis of Mango Callus After Agrobacterium Infection

**Figure 1:**
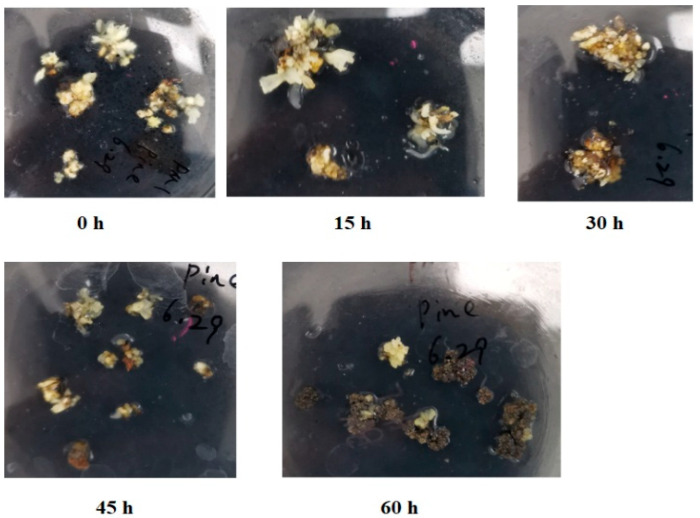
Mango callus infected with *Agrobacterium* for different time. 0 h, 15 h, 30 h, 45 h, and 60 h represented mango callus being infected by *Agrobacterium* for 0 h, 15 h, 30 h, 45 h, and 60 h, respectively. One single clone of *Agrobacterium* EHA105 was picked up from the YEB plate and cultured in liquid YEB at 26 °C for 48 h; 1 mL of *Agrobacterium* culture was put in fresh YEB liquid medium and cultured at 26 °C until OD_600_ = 1.0; 1.5 mL of *Agrobacterium* culture was centrifuged at 500 rpm. The supernatant was discarded. The pellet was resuspended with an AAM liquid medium containing 200 µmolL^−1^ acetosyringone and cultured in 500 mL of AAM liquid medium for 30 min. Ten grams of mango calluses were put into the triangular flask containing the *Agrobacterium* mixture and cultured for 30 min. And then, the mango calluses were put onto the sterile filter papers in the super-clean bench for 2 h to remove the water on the surface. After that, the mango calluses were transferred onto MS medium and cultured in 26 °C in dark for 0 h, 15 h, 30 h, 45 h, and 60 h, respectively.

When infected with *Agrobacterium,* most mango calluses turned brown and underwent necrosis within 60 h (Figure 1). In contrast, most of the uninfected calluses remained white or yellow. At 15 h post-infection (hpi), no browning or necrosis was observed in mango calluses. By 30 hpi, partial browning appeared in some calluses, accounting for approximately 7% of the total fresh weight. Browning and necrosis progressed rapidly by 45 hpi, with affected tissue increasing to 39% of the total callus mass. At 60 hpi, the majority of calluses exhibited severe browning and necrosis, constituting about 92% of the total fresh weight (Figure 1). After the browned and necrotized calluses were transferred onto a fresh medium and cultured for an additional two days, the browning and necrosis were not alleviated. The browning and necrosis continued until the calluses were necrotized completely. These demonstrated that *Agrobacterium* infection can induce irreversible browning and necrosis in mango callus, underscoring the urgent need for a solution.

### 2.2. Mango Callus Infected by Agrobacterium Contained More Reactive Oxygen Species (ROS) than Control

**Figure 2:**
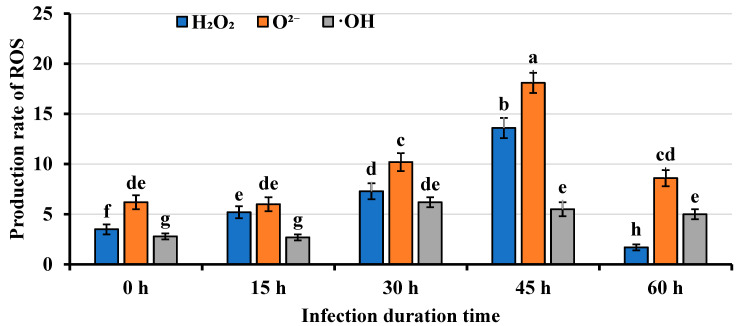
ROS contents in mango callus infected by *Agrobacterium*. The units for ROS levels are as follows: O^2−^, nmol/(min mg protein); H_2_O_2_, μmol/mg protein; ^•^OH, nmol/(min mg protein). 0 h, 15 h, 30 h, 45 h, and 60 h represented mango callus being infected by *Agrobacterium* for 0 h, 15 h, 30 h, 45 h, and 60 h, respectively. Different lowercase letters above the columns indicate significant differences between the treatments at the 0.05 level. The blue, orange, and grey columns represent H_2_O_2_, O^2−^, and ^•^OH content in mango callus, respectively. Each treatment consisted of four replicates. The significant differences between the treatments were tested by analysis of variance (ANOVA) and Duncan’s multiple range test (DMRT). SPSS 24 software was used to determine the significant difference at *p* ≤ 0.05. Data are presented as mean ± standard error (SE).

To investigate whether the browning and necrosis in *Agrobacterium*-infected mango callus infected by *Agrobacterium* was associated with ROS production, the levels of hydrogen peroxide (H_2_O_2_), superoxide anion (O^2−^), and hydroxyl radical (^•^OH) in the infected callus were measured. The results revealed a significant increase in ROS levels following *Agrobacterium* infection. At 15 h post-infection (hpi), H_2_O_2_ content in infected callus (5.2 ± 0.6 μmol/mg protein) was already significantly higher than in uninfected controls (3.5 ± 0.5 μmol/mg protein) (Figure 2). This increase continued over time, reaching 7.3 ± 0.8 μmol/mg protein at 30 hpi and peaking at 13.6 ± 1.0 μmol/mg protein at 45 hpi, followed by a gradual decline. Similarly, O^2−^ levels showed a comparable accumulation pattern. While no significant difference was observed between infected (6.0 ± 0.7 nmol/(min mg protein)) and uninfected callus (6.2 ± 0.7 nmol/(min mg protein)) at 15 hpi, the O^2−^ content increased markedly to 10.2 ± 0.9 nmol/(min mg protein) by 30 hpi and reached its maximum (18.1 ± 1.0 nmol/(min mg protein)) at 45 hpi before decreasing. The ^•^OH content exhibited a similar temporal pattern, with no significant difference at 15 hpi but reaching maximum levels at 30 hpi (Figure 2). Notably, all three ROS species maintained stable baseline levels in uninfected callus throughout the experimental period. These findings demonstrated that *Agrobacterium* infection triggered a rapid and substantial accumulation of ROS in mango callus, suggesting a potential role of oxidative stress in the browning and necrosis phenomena. The differential timing of peak accumulation among the various ROS species may reflect their distinct roles in the plant’s defense response.

### 2.3. Identification of Key Metabolites Associated with Browning in Mango Calluses Infected by Agrobacterium

**Figure 3:**
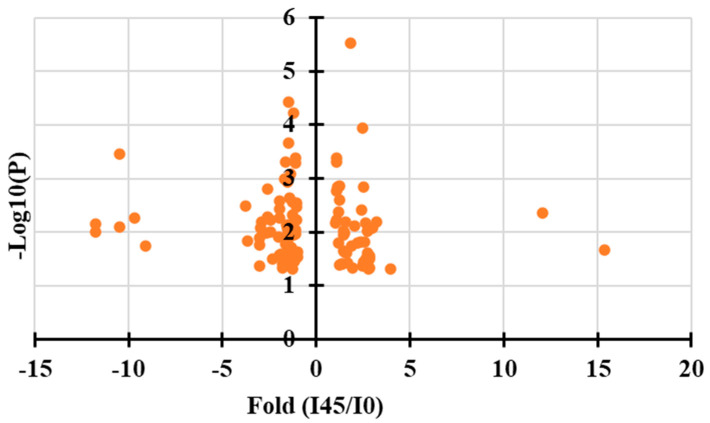
Quantitative plot of differentially expressed metabolites in callus infected by *Agrobacterium* for 45 h and uninfected.

To elucidate the metabolic basis of browning and necrosis in *Agrobacterium*-infected mango callus, a comparative metabolomic analysis between infected (45 hpi) and uninfected callus tissues was conducted. A total of 516 metabolites were detected in the samples. Using a threshold of fold-change >2.0 or <0.5 (infected vs. uninfected), 106 differentially accumulated metabolites, including 38 up-regulated and 68 down-regulated compounds, were identified (Figure 3). The seven most significantly up-regulated compounds were (in descending order of fold-change) gallic acid (15.2-fold increase), ubiquinone (11.5-fold), D-erythrose-4-phosphate, phosphoenolpyruvic acid, dihydrokaempferol, cyanidin, and dihydromyricetin. Variable Importance in Projection (VIP) scores further highlighted the significance of these metabolites (Table 1). Gallic acid exhibited both the highest fold-change (15.2) and VIP score (1.96) among all up-regulated metabolites, strongly suggesting its pivotal role in the browning and necrosis processes. The identified metabolites, particularly those involved in phenolic and flavonoid pathways (gallic acid, dihydrokaempferol, cyanidin), as well as key intermediates in energy metabolism (phosphoenolpyruvic acid, D-erythrose-4-phosphate), may collectively contribute to the observed physiological responses through their involvement in plant defense mechanisms and oxidative stress responses. These findings provided valuable insights into the metabolic change associated with *Agrobacterium* infection in mango callus, with gallic acid emerging as a potential key mediator of the browning phenotype.

### 2.4. The Expression Levels of ROS-Related Genes and Gallic Acid Synthesis Genes Were Up-Regulated in Callus Infected by Agrobacterium

To investigate the molecular mechanisms underlying immune responses in *Agrobacterium*-infected mango callus, transcriptome profiling of infected (45 hpi) and uninfected tissues was performed. Results showed that 1256 genes were up-regulated and 1528 down-regulated using the |log2FC| > 1 criteria and FDR < 0.05. Pathways were analyzed using the Kyoto Encyclopedia of Genes and Genomes (KEGG) database. Results showed that the up-regulated genes were mainly grouped in phenylpropanoid biosynthesis, phenylalanine metabolism, and antioxidant biosynthesis. The down-regulated genes were significantly enriched in photosynthesis, nucleic acid metabolism, DNA replication, and base excision repair. These demonstrated that after *Agrobacterium* infected mango callus, expressions of genes participating in phenylpropanoid biosynthesis, phenylalanine metabolism, and antioxidant biosynthesis were induced. The expressions of genes participating in photosynthesis, nucleic acid metabolism, DNA replication, and base excision repair were inhibited. The top 20 up-regulated genes are listed in Table 2. These genes mainly participated in gallic acid synthesis (*MiDAHPS1*, *MiDHQD1*, *MiDHQD2*, *MiSHD1*, *MiSHD2*), ROS biosynthesis (*MiCAT1*, *MiCAT2*, *MiGOX1*, *MiGOX2*, *MiFOX1*, *MiFOX2*, *MiNADO1*, *MiNADO2*), and defense reactions (*MiFLS2-1*, *MiMPK3-1*, *MiMPK6-1*, *MiWRKY22-1*, *MiNHL10-1*) (Table 2). These findings demonstrated that *Agrobacterium* infection triggers a coordinated transcriptional reprogramming in mango callus, characterized by activation of phenolic compound biosynthesis (particularly gallic acid pathway), induction of ROS-generating and scavenging systems, up-regulation of defense signaling components, and concurrent suppression of growth-related processes. The simultaneous up-regulation of gallic acid biosynthesis genes and ROS-related enzymes suggests their potential synergistic role in the browning phenotype and defense responses against *Agrobacterium* infection.

### 2.5. Validation of Immune-Related Gene Expression Patterns in Agrobacterium-Infected Mango Callus

To confirm the transcriptome findings, quantitative real-time PCR (qRT-PCR) analysis on partially up-regulated genes was conducted. The qRT-PCR results strongly corroborated the transcriptome data, demonstrating high consistency in expression patterns between both methodologies (Table 3). For example, the transcriptome experiment showed that the expression of *MiDAHPS1* in mango callus infected by *Agrobacterium* for 45 h was 5.2-fold of that in callus uninfected (Table 2). QRT-PCR results showed that transcripts of *MiDAHPS1* in mango calluses infected by *Agrobacterium* for 45 h were 5-fold of those in calluses uninfected (Table 3). Expressions of other up-regulated genes between transcriptome and qRT-PCR also showed similar trends (Table 2 and Table 3). Furthermore, the changing trends of the expressions of the up-regulated genes were highly consistent with those of the ROS contents in mango calluses infected by *Agrobacterium*. For example, the contents of H_2_O_2_, O^2−^, and ^•^OH in mango callus increased after the callus was infected by *Agrobacterium* (Figure 2). ROS contents in the callus reached their most after the callus was infected for 45 h (Table 3), and then, they decreased (Figure 2). Transcripts of the up-regulated genes using qRT-PCR showed the same trends (Table 3). For example, the expression of *MiDAHPS1* in callus infected for 45 h was the highest among the five treatments (Table 3). Expression of *MiDAHPS1* in callus infected for 60 h was less than that in callus infected for 45 h (Table 3). The changing trends of the expressions of genes participating in ROS biosynthesis, defense responses, and gallic acid biosynthesis were consistent with those of the ROS contents in the callus and those of the callus browning and necrosis. These genes essentially participated in the immune responses in mango calluses infected by *Agrobacterium*. These results validate that *Agrobacterium* infection triggers a coordinated transcriptional reprogramming in mango callus, characterized by temporally coupled activation of ROS metabolic pathways, phenolic compound (particularly gallic acid) biosynthesis, and defense signaling cascades. The correlation between molecular responses (gene expression), biochemical changes (ROS levels), and phenotypic manifestations (browning/necrosis) strongly supports the conclusion that these pathways constitute integral components of the mango callus immune response to *Agrobacterium* infection.

### 2.6. The Inhibitory Effect of Gallic Acid on Agrobacterium Growth

**Figure 4:**
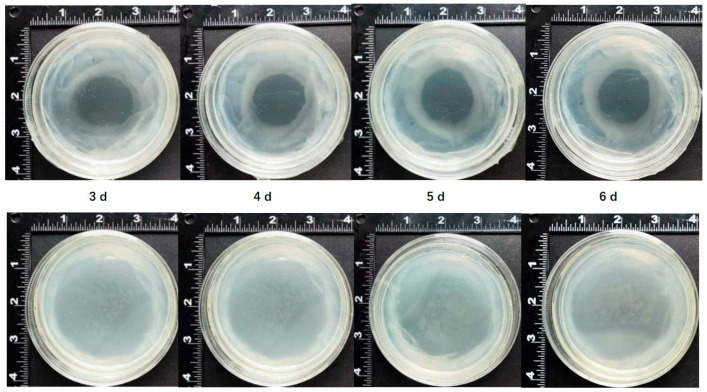
Gallic acid inhibits the growth of *Agrobacterium.* One single clone of *Agrobacterium* EHA105 was picked up from the YEB plate and cultured in liquid YEB at 28 °C for 48 h. One milliliter of *Agrobacterium* cultures was put in fresh YEB liquid medium and cultured at 26 °C until OD_600_ = 1.0. Five hundred microliters of *Agrobacterium* cultures were spread onto the MS plate. One hundred microliters of gallic acid (10 mg/L) were pipetted onto the plate center. MS plates without gallic acid were used as a control. The MS plates were incubated at 28 °C for 3, 4, 5, and 6 days in the dark, respectively. The top four plates showed *Agrobacterium* grown on an MS medium on which 100 μL of 10 mg/L gallic acid was pipetted onto the center. The bottom four plates showed *Agrobacterium* grown on medium without gallic acid; 3 d, 4 d, 5 d, and 6 d represented MS plates cultured at 28 °C for 3, 4, 5, and 6 days, respectively.

Both metabolome and transcriptome experiments showed that gallic acid participated in immune responses in mango calluses infected by *Agrobacterium* (Table 1 and Table 2). To study the relationship between gallic acid and the immune response of mango callus infected by *Agrobacterium*, 100 μL of *Agrobacterium* culture was spread on an MS plate. One hundred microliters of 10 mg/L gallic acid was pipetted onto the center of the plate. The MS plate was put in an incubator at a temperature of 28 °C for 3–6 days. Results showed that after *Agrobacterium* was cultured for 3 days, there was an empty circular area on the plate (Figure 4). *Agrobacterium* cannot propagate in this circular area. After *Agrobacterium* was cultured for 4 d, 5 d, or 6 d, more *Agrobacterium* cells accumulated outside the circular area (Figure 4). Especially for MS plates pipetted with gallic acid and cultured for 5 d and 6 d, a dense *Agrobacterium* band can be found outside and close to the circle (Figure 4). However, no *Agrobacterium* cell was found in the circular area (Figure 4). These demonstrated that *Agrobacterium* cells cannot grow on the MS plate area pipetted with gallic acid. After being cultured for a longer time, although more *Agrobacterium* cells accumulated in the MS plate area without gallic acid, they did not grow in the field containing gallic acid. These indicated that gallic acid can inhibit the growth of *Agrobacterium,* which might decrease the efficiency of *Agrobacterium* infection.

### 2.7. Suppression of Immune Responses in Mango Callus by Agrobacterium (T3SS-AvrPto)

**Figure 5:**
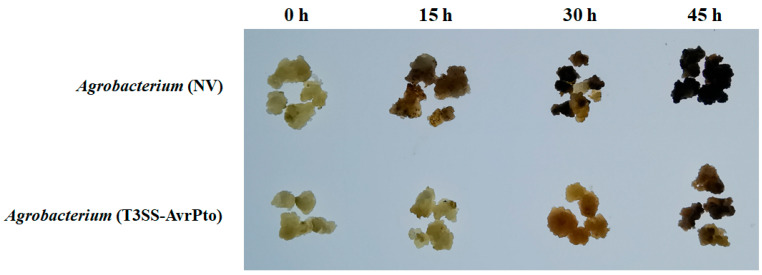
The browning and necrosis of callus infected by *Agrobacterium.* The 0 h, 15 h, 30 h, and 45 h represented mango calluses cultured for 0 h, 15 h, 30 h, and 45 h, respectively, after being infected by *Agrobacterium*. One single clone of *Agrobacterium* was picked up from the YEB plate and cultured in liquid YEB at 26 °C for 48 h; 1 mL of *Agrobacterium* culture was put in fresh YEB liquid medium and cultured at 26 °C until OD_600_ = 1.0; 1.5 mL of *Agrobacterium* culture was centrifuged at 500 rpm. The supernatant was discarded. The pellet was resuspended with an AAM liquid medium containing 200 µmolL^−1^ acetosyringone and cultured in 500 mL of AAM liquid medium for 30 min. Ten grams of mango calluses were put into the triangular flask containing the *Agrobacterium* mixture and cultured for 30 min. And then, the mango calluses were put onto the sterile filter papers in the super-clean bench for 2 h to remove the water on the surface. After that, the mango calluses were transferred onto MS medium and cultured in 26 °C in dark for 0 h, 15 h, 30 h, and 45 h, respectively.

To study whether T3SS and its effector protein can inhibit the immune response in mango callus induced by *Agrobacterium*, *P. syringae* pv. tomato strain DC3000 T3SS gene cluster (GenBank accession number: AF232004.3), effector gene *AvrPto* (NC_004578.1), and its promoter (116 bp upstream of ATG) were transformed into *Agrobacterium* EHA105 in this research. The transformed *Agrobacterium* was named *Agrobacterium* (T3SS-*AvrPto*). *Agrobacterium* transformed with a net vector was named *Agrobacterium* (NV). Mango callus was infected with *Agrobacterium* (T3SS-*AvrPto*) and *Agrobacterium* (NV), respectively, according to the method described in the Materials and Methods Section 4. The callus browning and the necrosis process were monitored. Results showed that after being infected by *Agrobacterium* (NV) for 15 h, about 30% of the calluses were browned and necrotized (Figure 5). After being infected with *Agrobacterium* (NV) for 30 h, about 90% of the calluses were browned and necrotized (Figure 5). If mango callus was infected with *Agrobacterium* (T3SS-*AvrPto*) for 15 h, a few calluses were browned or necrotized (Figure 5). When the callus was infected with *Agrobacterium* (T3SS-*AvrPto*) for 45 h, only about 10% of the calluses were browned (Figure 5). Furthermore, most of the browned calluses did not necrotize (Figure 5). Mango calluses infected by *Agrobacterium* (T3SS-*AvrPto*) browned and necrotized remarkably slower than those infected by *Agrobacterium* (NV) (Figure 5). These indicated that mango calluses infected by *Agrobacterium* (T3SS-*AvrPto*) had less immune response than those infected with *Agrobacterium* (NV).

### 2.8. Mango Callus Infected by Agrobacterium (T3SS-AvrPto) Produced Less ROS than Those Infected by Agrobacterium (NV)

To identify whether the mango callus infected by *Agrobacterium* (T3SS-*AvrPto*) browned and necrotized slower than *Agrobacterium* (NV) was due to that the callus infected by *Agrobacterium* (T3SS-*AvrPto*) had weaker immune responses, ROS in calluses infected by *Agrobacterium* (T3SS-*AvrPto*) and those infected by *Agrobacterium* (NV) were determined. Results showed that after the mango calluses were infected for 15 h, H_2_O_2_ content in the calluses infected by *Agrobacterium* (NV) was 5.0 ± 0.6 μmol/mg protein, while that in calluses infected by *Agrobacterium* (T3SS-*AvrPto*) was 3.9 ± 0.5 μmol/mg protein (Table 4). The H_2_O_2_ content in the calluses infected by *Agrobacterium* increased with more time, and the H_2_O_2_ content in the callus reached the maximum when the callus was infected for 45 h, and then decreased. At every time point, H_2_O_2_ content in mango callus infected by *Agrobacterium* (T3SS-*AvrPto*) was significantly less than that in callus infected by *Agrobacterium* (NV) (Table 4). Except for 15 h, O^2−^ content and ^•^OH content in mango callus infected by *Agrobacterium* (T3SS-*AvrPto*) were less than those in callus infected by *Agrobacterium* (NV), correspondingly (Table 4). ROS contents in mango calluses infected by *Agrobacterium* (T3SS-*AvrPto*) were less than those in calluses infected by *Agrobacterium* (NV) (Table 4). These demonstrated that mango calluses infected by *Agrobacterium* (T3SS-*AvrPto*) have weaker immune responses than those infected by *Agrobacterium* (NV), resulting in calluses infected by *Agrobacterium* (T3SS-*AvrPto*) browned and necrotized slower than those infected by *Agrobacterium* (NV).

### 2.9. Suppression of Immune Responses in Mango Callus Infected by Agrobacterium (T3SS-AvrPto) Compared to Agrobacterium (NV)

To study the mechanism underlying the phenomenon that mango callus infected by *Agrobacterium* (T3SS-*AvrPto*) had less immune response than callus infected by *Agrobacterium* (NV). The expressions of the top up-regulated genes in calluses infected by *Agrobacterium* were measured in mango calluses infected by *Agrobacterium* (T3SS-*AvrPto*) and those infected by *Agrobacterium* (NV) using qRT-PCR. Results showed that the expressions of these genes in calluses infected by *Agrobacterium* (T3SS-*AvrPto*) for 45 h were significantly less than those in calluses infected by *Agrobacterium* (NV) for 45 h (Table 5). At 15 h, 30 h, and 60 h, the expressions of these genes in calluses infected by *Agrobacterium* (T3SS-*AvrPto*) were less than or similar to those in calluses infected by *Agrobacterium* (NV) (Table 5). For example, the relative expression of *MiDAHPS1* in calluses infected by *Agrobacterium* (T3SS-*AvrPto*) for 45 h was 2.0 ± 0.2 (Table 5). The value in calluses infected by *Agrobacterium* (NV) for 45 h was 5.1 ± 0.3 (Table 5). Relative expression of MiDAHPS1 in calluses infected by *Agrobacterium* (T3SS-*AvrPto*) for 45 h was significantly less than that in calluses infected by *Agrobacterium* (NV) for 45 h (Table 5). Relative expression of *MiDHQD1* in calluses infected by *Agrobacterium* (T3SS-*AvrPto*) for 15 h was 0.5 ± 0.1 (Table 5). The value in callus infected by *Agrobacterium* (NV) for 15 h was 0.6 ± 0.1 (Table 5). Relative expression of *MiDHQD1* in calluses infected by *Agrobacterium* (T3SS-*AvrPto*) was similar to that in calluses infected by *Agrobacterium* (NV) for 15 h (Table 5). These indicated that when the mango callus was infected by *Agrobacterium* (T3SS-*AvrPto*), the expression of the genes up-regulated during immune responses was either inhibited or maintained. Immune reactions in calluses infected by *Agrobacterium* (T3SS-*AvrPto*) were suppressed.

### 2.10. Agrobacterium Harboring Type III Secretion Gene Cluster (T3SS) and Effector Gene AvrPto Had Higher Infection Efficiency than Control Agrobacterium

**Figure 6:**
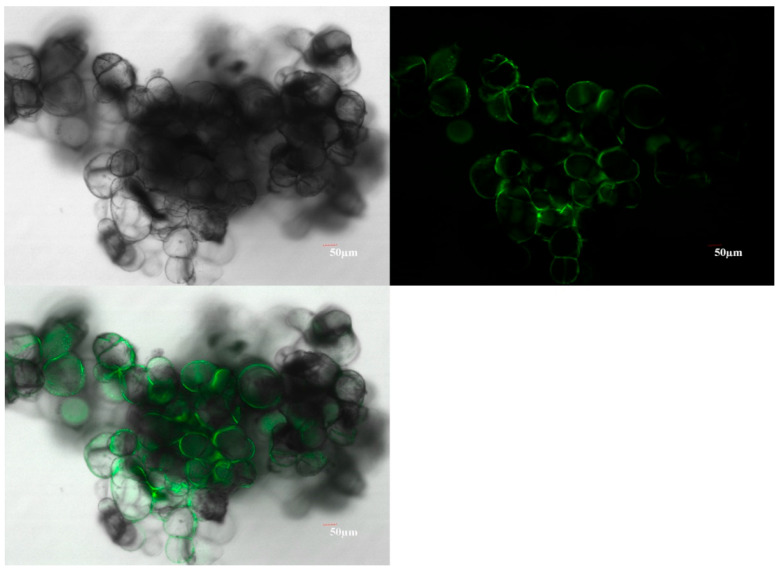
EGFP fluorescence observation in mango calluses infected with *Agrobacterium* (T3SS-*AvrPto*-EGFP). The top-left panel represented EGFP fluorescence observed in mango calluses in bright fields. The top-right panel represented EGFP fluorescence observed in mango calluses in dark fields. The bottom panel represented EGFP fluorescence observed in mango calluses in the fused field.

**Figure 7:**
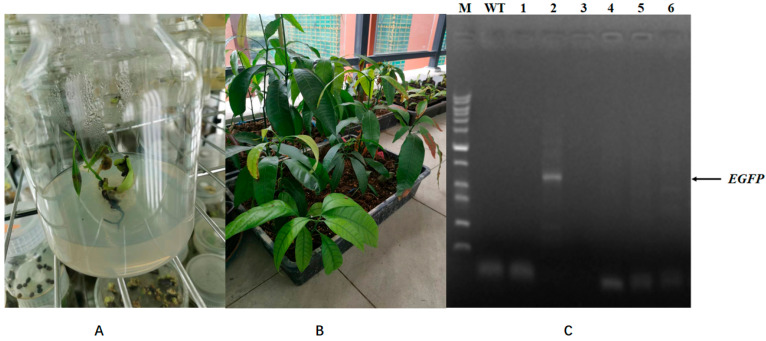
Differentiation of the transgenic calluses and verification. (**A**) The possible transgenic seedling grown on media containing 20 μg/mL chloromycetin. (**B**) The possible transgenic seedlings grown in gardening soil. (**C**) Verification of the seedlings differentiated. Genomic DNA was extracted from the leaves of the possible transgenic plants. PCR was performed using P1F:5′-ATGGTGAGCAAGGGCGAGGA-3 and P1R:5′-CTTGTACAGCTCGTCCATGC-3′ as primers responding to the EGFP gene in the plasmid pBI221-GFP. 1–6 represented different seedlings. WT referred to mango seedlings differentiated from calluses not infected by *Agrobacterium*.

To study whether the transgenic efficiency of mango calluses can be improved using *Agrobacterium* harboring a type III secretion gene cluster (T3SS) and effector gene *AvrPto*, *Agrobacterium* (T3SS-*AvrPto*-EGFP) and *Agrobacterium* (NV-EGFP) were constructed as described in Materials and Methods. Mango calluses were infected with *Agrobacterium* (T3SS-*AvrPto*-EGFP), and EGFP fluorescence in calluses was observed. Results showed that about 1.6% of calluses infected by *Agrobacterium* (T3SS-*AvrPto*-EGFP) had successfully been transformed (Figure 6). No EGFP fluorescence was found in the callus infected with *Agrobacterium* (NV-EGFP). Mango calluses infected by *Agrobacterium* (T3SS-*AvrPto*-EGFP) had remarkably higher transgene efficiency than those infected by *Agrobacterium* (NV-EGFP). *Agrobacterium*-harboring T3SS-*AvrPto* had higher infection efficiency than *Agrobacterium* (NV). After the infected calluses were cultured on medium containing 20 μg/mL chloromycetin and 400 mg/L timentin for 1 month, the transformed calluses differentiated into seedlings. The possible transgenic seedlings were identified using PCR. Results showed that some seedlings had been transformed successfully (Figure 7). These findings underscore the potential of inhibiting the immune responses in mango calluses to enhance their transgene efficiency and the possibility of improving the transgene efficiency of mango calluses by constructing an *Agrobacterium* strain harboring T3SS and *AvrPto*.

## 3. Discussion

After multiple infections of the mango embryogenic callus using *Agrobacterium tumefaciens*, we observed that the majority of the callus rapidly browned and necrotized post-infection. The surviving callus eventually died during the differentiation culture process, and no regenerated plants were obtained. When pathogenic bacteria infect plants, the plants trigger immune responses, leading to programmed cell death to prevent the spread of the pathogen within the host [16,17]. The infection of mango embryogenic callus by *Agrobacterium tumefaciens* may have similarly induced such immune responses. Suppressing these immune reactions could significantly improve the transgenic efficiency of mango embryogenic calluses.

The secretion system of *Agrobacterium tumefaciens* is a type IV secretion system (T4SS), which facilitates the extracellular delivery of bacterial effector proteins [18]. However, many plants exhibit immune responses against T4SS and *Agrobacterium* effector proteins [19]. For example, *Pseudomonas syringae* employs a type III secretion system (T3SS) to translocate its effector proteins. *Agrobacterium* transformed with the T3SS gene cluster and the effector gene *AvrPto* from *P. syringae* can escape being perceived by plant cells [15]. Using the engineered *Agrobacterium*, the transformation rate of wheat, alfalfa, and switchgrass increased by 250–400% [15]. The transformation rate of mango calluses infected with *Agrobacterium* (T3SS-*AvrPto*-EGFP) was about 1.6%, which is a promising finding for the potential application of the transgenic method in mango breeding. This strategy can also be used for constructing stable transgenic plants of recalcitrant species.

ROS can injure or kill plant cells, inducing callus browning and necrotized. High content of ROS induced plant cells to necrotize and die [20]. Immune response can be triggered by ROS (Tang et al., 2017) [21]. After the grape embryogenic calluses were infected by *Agrobacterium*, key ROS-removal enzymes were down-regulated and ROS-production-related proteins were up-regulated [22]. H_2_O_2_ concentration and peroxidase activity increased in the callus [22]. The expression of ascorbate peroxidase (APX) decreased. APX is involved in the cell response to oxidative stress and plays a key role in regulating H_2_O_2_ levels in plants [22]. We found that after being infected by *Agrobacterium* for 15 h, H_2_O_2_ content in mango callus was 5.2 ± 0.6 μmol/mg protein. H_2_O_2_ content in uninfected mango calluses was 3.5 ± 0.5 μmol/mg protein. After the callus was infected for 45 h, H_2_O_2_ content in the mango callus was 13.6 ± 1.0 μmol/mg protein. O^2−^ in calluses infected by *Agrobacterium* at different time points showed a similar trend. ^•^OH content in calluses infected for 15 h was similar to that in uninfected calluses. After being infected for 30 h, ^•^OH content in the callus reached the maximum. ROS content in the calluses increased rapidly after they were infected by *Agrobacterium*. Among the 20 top up-regulated genes in the mango callus, *MiCAT1*, *MiCAT2*, *MiGOX1*, *MiGOX2*, *MiFOX1*, *MiFOX2*, *MiNADO1*, and *MiNADO2* participated in ROS biosynthesis. These indicated that ROS played important roles in inhibiting mango transformation upon being infected by *Agrobacterium*.

Darkening of explants frequently observed during *Agrobacterium*-mediated plant transformation has also generally been attributed to phenolic production, which may eventually lead to the death of plant cells [23,24,25]. When plant cells were injured, phenolic compounds in the cells were oxidized, and ubiquinone formed [26]. Ubiquinone was the brown compound in the callus [27]. Dihydrotricetin, dihydrokaempferol, and cyanidin were all phenolic compounds. When the mango callus was infected by *Agrobacterium*, the contents of dihydrotricetin, dihydrokaempferol, and cyanidin in the mango callus increased. More ubiquinone will be synthesized in the callus, and the callus will brown, as shown in the metabolome experiment in this research.

DAHPS, DHQD, and SDH participated in gallic acid biosynthesis [28]. In this research, it was found that the expressions of these genes were up-regulated after *Agrobacterium* infected the callus. This might improve the synthesis of gallic acid, as seen in the metabolome experiment in this research. Gallic acid is a common phenolic acid widely used as an antibacterial agent in the food industry [29]. It is recognized as a compound with broad-spectrum antibacterial and long-lasting sterilizing properties [29]. This research showed that *Agrobacterium* did not grow on an MS plate containing gallic acid. The growth of *Agrobacterium* cells can be inhibited by gallic acid. Failure of the mango transgene might be partially due to the gallic acid produced in the callus. If the reagents that can degrade gallic acid or inhibit the function of gallic acid were added to the liquid medium when mango calluses were infected by *Agrobacterium*, more *Agrobacterium* cells would survive, and more mango calluses would be infected. The infection efficiency of the *Agrobacterium* and the transformation rate of the mango calluses might also be enhanced. These are worth being investigated in the future.

After the grape embryogenic callus was infected by *Agrobacterium*, PR10 was one of the most strongly up-regulated proteins [22]. PR10 was a protein with a role in the plant defense reaction [22]. In this study, after mango calluses were infected with *Agrobacterium*, the expressions of *MiFLS2-1*, *MiMPK3-1*, *MiMPK6-1*, *MiWRKY22-1*, and *MiNHL10-1* increased significantly. They all participated in plant defense reactions, demonstrating that immune responses and defense reactions played key roles in the browning and necrosis of mango callus after being infected by *Agrobacterium*.

**Figure 8:**
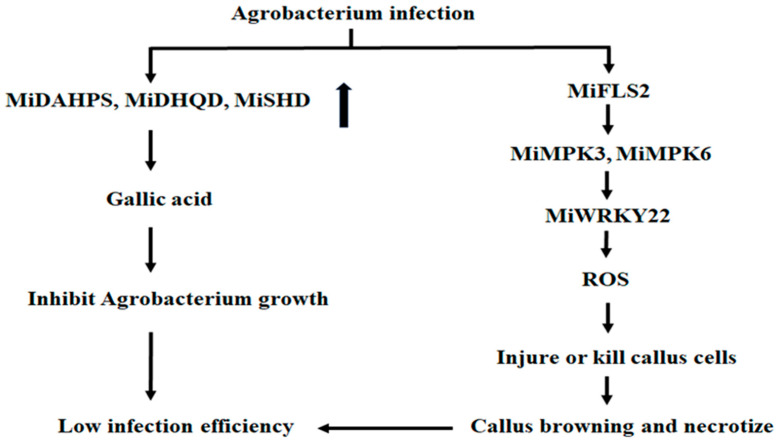
The proposed mechanism underlying the immune response of mango calluses infected by *Agrobacterium*. The boldfaced arrow represented the expressions of the up-regulated genes.

Pattern-recognized receptors (PRRs) on the plant cell membranes can perceive pathogens and activate the immune system of plant cells [30]. Arabidopsis FLAGELIN-SENSITIVE2 (FLS2) was an identified PRR, which can perceive the 22 conserved amino acids in the N-terminal of bacterial flagellin (flg22) and interact with them [31]. After perceiving flg22, FLS2 combined with Brassinosteroid receptor-associated kinase 1 (BAK1) and formed a complex, which activated the downstream mitogen-activated protein kinase 3 (MPK3) and mitogen-activated protein kinase 6 (MPK6) [32]. MPK3 and MPK6 induced the expression of WRKY22, WRKY29, FRK1 (FLG22-induced receptor-like kinase 1), and other defense-related genes, producing more ROS [32]. The high content of ROS induced necrosis and death in plant cells [26]. Arabidopsis EF-Tu receptor (EFR) was also a PRR [33]. EFR can perceive *Agrobacterium* elongation factor Tu (EF-Tu) and activate plant immune response [34]. The polypeptide constituted by 18 amino acids located in the N-terminal of EF-Tu (elf18) can induce ROS production quickly, which causes the expression of defense genes FRK1, NHL10 (NDR1/HIN1-like 10), and so on [35]. It was found that the expressions of *MiFLS2-1*, *MiMPK3-1*, *MiMPK6-1*, *MiWRKY22-1*, and *MiNHL10-1* in mango calluses infected by *Agrobacterium* were all higher than those in uninfected calluses, suggesting that when *Agrobacterium* infected calluses, these proteins were synthesized rapidly, which further induced the expression of the ROS-related genes. ROS was quickly produced. Some or all of the callus was damaged or killed by excessive ROS. Some infected calluses browned, and some necrotized, resulting in a low transformation rate (Figure 8). Gallic-acid synthesis genes and defense-related genes constituted the immune response system in the mango callus (Figure 8). This differed from Arabidopsis, which was caused only by defense-related genes. This is probably why the efficiency of conventional *Agrobacterium* infection on the mango callus was low, and the mango transgene experiment was challenging.

## 4. Materials and Methods

### 4.1. Agrobacterium Strain

The *Agrobacterium* strain used in this research was EHA105, which is a derivative of the soil bacterium *Agrobacterium tumefaciens*, widely used in plant genetic engineering due to its ability to transfer foreign DNA into plant cells. It is a disarmed strain, meaning its tumor-inducing (Ti) plasmid has been modified to remove pathogenic genes while retaining the DNA transfer mechanism essential for genetic transformation. EHA105 is derived from the wild-type strain *A. tumefaciens* C58, specifically from its helper strain EHA101. The Ti plasmid (pTiBo542) is disarmed by removing oncogenes (e.g., iaaM, ipt) but retaining virulence (vir) genes required for T-DNA transfer. A binary vector system: A separate plasmid (e.g., pCAMBIA series) carries the gene of interest and T-DNA borders, while the disarmed Ti plasmid provides vir genes in trans. *Agrobacterium* EHA105 is non-pathogenic, which cannot cause crown gall disease due to the absence of oncogenes. It has high transformation efficiency for dicotyledonous plants and some monocots.

### 4.2. Construction of Agrobacterium Strains

The DNA fragment containing the *P. syringae* pv. tomato strain DC3000 T3SS gene cluster (GenBank accession number: AF232004.3), effector gene *AvrPto* (NC_004578.1), and its promoter (116 bp upstream of ATG) was meticulously synthesized with attB sequences at the two terminals. The BP recombination reaction was carried out with the same level of meticulousness, inserting the synthesized DNA into pENTRTM5′-TOPO (Invitrogen, Carlsbad, CA, USA) using the pENTRTM5′-TOPO Cloning Kit (Invitrogen). *E. coli* TOP10 was used to propagate the plasmid. The plasmids pENTRTM5′-TOPO-T3SS-*AvrPto* were extracted using the GeneJET kit (Thermo Fisher Scientific, Waltham, MA, USA). The LR recombination reaction was performed with the same level of meticulousness, transferring T3SS-*AvrPto* in pENTRTM5′-TOPO-T3SS-*AvrPto* into pEarleyGate302 (BIOESN, Shanghai, China) using the LR Clonase™ Enzyme Mix (Invitrogen). *E. coli* TOP10 was used to propagate the plasmid. The plasmids pEarleyGate302-T3SS-*AvrPto* were extracted and transformed into *Agrobacterium* EHA105. Triparental mating [36] was used to mobilize pEarleyGate302-T3SS-*AvrPto* into *A. tumefaciens* EH105. *E. coli* HB101 harboring the helper plasmid pRK2013 was bought from Beijing Biobw Biotechnology Co., Ltd. (Beijing, China) (Bio-133891) [37,38]. A single clone of *E. coli* DH10B harboring pEarleyGate302-T3SS-*AvrPto* was picked up from the LB plate and cultured in LB liquid medium containing kanamycin until OD_600_ = 0.5–0.8. *E. coli* HB101 harboring the helper plasmid pRK2013 was also cultured in LB liquid medium containing 50 μg/mL kanamycin until OD_600_ = 0.5–0.8. *Agrobacterium* EHA105 was cultured in YEB liquid medium containing 100 μg/mL rifampicin until OD_600_ = 0.5–0.8. One hundred and fifty microliters of the 3 cultures were mixed and centrifuged at 800 rpm for 20 min. The supernatant was discarded, and the pellet was resuspended in 300 μL LB liquid medium. The mixture was spread on a YEB plate containing 50 μg/mL kanamycin and 100 μg/mL rifampicin. The plate was put at 28 °C for 24 h. Single clones were picked up and cultured in YEB liquid medium containing 50 μg/mL kanamycin and 100 μg/mL rifampicin, and PCR was performed. P2F: 5′-GGGGACAAGTTTGTACAAAAAAGCAGGCT-3′, P2R: 5′-CTACCCATACGATGTTCCAG-3′. Clones with the expected PCR result were stocked to infect mango calluses. To construct *Agrobacterium* (T3SS-*AvrPto*-EGFP), *Agrobacterium* (T3SS-*AvrPto*) was transformed with plasmid pBI221-GFP (HonorGene, Changsha, China), and the transformed *Agrobacterium* was screened on a plate containing 100 μg/mL ampicillin and 100 μg/mL rifampicin.

### 4.3. Mango Embryogenic Callus Induction

Mango callus was induced and modified according to the published method [39]. Fruitlet in the cotyledon stage (45 days after the flower) was collected. Contaminants on the surface were removed using a brush. The fruit was rinsed for 30 min using tap water. It was transferred into 1% detergent and marinated for 20 min. The fruitlet was washed using flush water for 20 min. 75% ethanol was sprayed onto the surface. The fruitlet was cut using a scalpel in the super clean bench, and the seed was fetched. The seed was longitudinally cut. The embryonic matter was collected from the inner surface of the seed and cultured on medium M1 (Table 6). The plates were put in the dark at 26 °C for one month. And then, the callus induced was transferred onto medium M2 (Table 6). The plates were put in a dark place at 26 °C and cultured for one month. A large amount of embryogenic callus will be obtained.

### 4.4. Mango Callus Transformation

One gram of mango embryogenic callus is placed on a medium containing chloromycetin concentrations. The plates are then kept in the dark at 25 °C for one month to identify the concentration at which the callus cannot grow. *Agrobacterium* cells transformed with pEarleyGate302-T3SS-*AvrPto* or pBI221-GFP are cultured overnight; 1.5 mL of the *Agrobacterium* culture is centrifuged at 500 rpm, and the supernatant is discarded. The pellet is resuspended with an AAM liquid medium containing 200 µmolL^−1^ acetosyringone and shaken at 100 rpm, 26 °C for 1 h. The mango callus is placed in the AAM medium and cultured for 30 min. The liquid is discarded, and the callus is put on sterilized filter paper on a super-clean bench for 60 min. Next, the callus is transferred onto medium M3 (Table 6). The plates are observed in the dark at 26 °C for 30 days.

### 4.5. Metabolome and Transcriptome Experiments

Metabolome and transcriptome experiments were performed as described before [40]. *Agrobacterium* was cultured at 26 °C in the dark for two days. The OD_600_ was adjusted to 0.4; 1.5 mL of *Agrobacterium* culture was collected and centrifuged at 500 rpm. The supernatant was discarded. The pellet was resuspended in 1 mL AAM medium containing 200 µmolL^−1^ acetosyringone. The mixture was transferred into 100 mL AAM medium containing 200 µmolL^−1^ acetosyringone and cultured for 1 h in the dark at 26 °C; 10 g of mango embryogenic callus was put in AAM medium and shaken at 500 rpm, 26 °C in the dark for 30 min. The liquid was discarded. The callus was washed with sterilized water ten times. The callus was stored at −80 °C until metabolome and transcriptome experiments were performed.

For analyzing the metabolome results, unsupervised PCA (principal component analysis) was performed by the statistics function prcomp within R (www.r-project.org, accessed on 6 May 2023). The data were unit variance scaled before unsupervised PCA. The HCA (hierarchical cluster analysis) results of samples and metabolites were presented as heatmaps with dendrograms, while Pearson correlation coefficients (PCC) between samples were calculated by the cor function in R and presented as only heatmaps. Both HCA and PCC were carried out by the R package pheatmap. For HCA, normalized signal intensities of metabolites (unit variance scaling) are visualized as a color spectrum. Significantly regulated metabolites between groups were determined by VIP ≥ 1 and absolute Log2FC (fold change) ≥ 1. VIP values were extracted from the OPLS-DA result, which also contains score plots and permutation plots, and was generated using the R package MetaboAnalystR 3.0. The data were log-transformed (log2) and mean-centered before OPLS-DA. In order to avoid overfitting, a permutation test (200 permutations) was performed. Identified metabolites were annotated using the KEGG Compound database (http://www.kegg.jp/kegg/compound/, accessed on 10 May 2023); annotated metabolites were then mapped to the KEGG Pathway database (http://www.kegg.jp/kegg/pathway.html, accessed on 8 July 2023). Pathways with significantly regulated metabolites mapped were then fed into MSEA (metabolite sets enrichment analysis); their significance was determined by the hypergeometric test’s *p*-values.

### 4.6. QRT-PCR

NucleoSpin(R) 96 RNA core kit (Thermo Fisher Scientific, Waltham, MA, USA) was used to extract mango callus RNA. RNA was transcribed into cDNA using Oligo(dT) 12–18 and SuperScript III Reverse Transcriptase (Invitrogen). QRT-PCR was performed using a CFX Real-time PCR system (Applied Biosystems, Foster City, CA, USA) and KiCqStart SYBR Green qPCR ReadyMix (Catalog No. KCQS01, MilliporeSigma, Burlington, MA, USA). QRT-PCR data were collected using the software Bio-Rad CFX Manager Version 2.1.1022.0523. The relative expression was calculated using 2^−ΔΔCT^. Housekeeping gene tubulin (XM_044625649.1) was used as a control. Genomic DNA in RNA samples was removed using TURBO DNase (Catalog No. AM1907, Invitrogen). The raw Ct values were transformed into relative quantities or copy numbers. The target gene Ct values were normalized using the reference gene (tubulin, XM_044625649.1): ΔCt = ΔCt_target gene_ − ΔCt_reference gene_. A curve was generated using standards of known concentrations to calculate absolute copy numbers. If the data follow an exponential distribution, a log transformation (log2) was applied to stabilize variance. ΔΔCt was calculated, and ΔCt values between experimental and control groups were compared: ΔΔCt = ΔCt_experimental group_ − ΔCt_control group_. Fold change was calculated as follows. Fold Change = 2^−ΔΔCt^ (assuming 100% amplification efficiency). The Shapiro–Wilk test was used to assess whether the data follow a normal distribution. If it were a normal distribution, ANOVA was applied to compare differences among multiple groups, followed by post-hoc tests (Tukey’s HSD). If it were non-normally distributed, the Kruskal–Wallis test was used for comparisons among multiple groups. Bonferroni correction was applied to control the false discovery rate.

### 4.7. Confocal Observation

Green fluorescence in transformed callus was observed using confocal, as described in a published paper [41]. The exciting light wavelength was 488 nm. The scattered light wavelength was 493–550 nm. The eyepiece magnification is 10×, and the objective lens magnification is 20×. The results were analyzed using the software Leica Application Suite X (LAS X) version 3.5.5.19976.

### 4.8. Determination of ROS Contents in Mitochondria Isolated from Mango Callus

According to the published paper, mitochondria were isolated and purified from mango calluses [42]. The mitochondrial pellets were resuspended in a buffer (20% glycerol, 0.1 M Tris, pH 7.0). The contents of H_2_O_2_ and O^2−^ were measured using established methods [43], and the ^•^OH content was determined as previously described [44]. A 0.5 mL suspended mitochondrial buffer was added to a 0.5 mL basic reaction system (120 mmolL^−1^ KCl, 0.4 molL^−1^ NADH, 30 mmolL^−1^ DMSO, 40 mmolL^−1^ phosphate buffer solution, pH 7.0). The mixture was then incubated at 25 °C for 10 min. The production of formaldehyde was calculated from the Nash reaction to determine the content of ^•^OH [45].

### 4.9. Statistical Analysis

Each treatment consisted of three replicates. The significant differences between the treatments were tested by analysis of variance (ANOVA) and Duncan’s multiple range test (DMRT). SPSS software was used to determine the significant difference at *p* ≤ 0.05. Data are presented as mean ± standard error (SE).

## 5. Conclusions

The low efficiency of the mango transgene was due to immune responses activated in the callus. The infection and transformation efficiency can be significantly improved if the mango calluses are infected by *Agrobacterium* harboring a T3SS gene cluster and its effector gene. The transformation rate of mango callus can be enhanced to 1.6% when they are infected with *Agrobacterium* (T3SS-*AvrPto*), which is a promising finding. These may play important roles in developing new mango varieties with multiple resistance traits in the future.

## Figures and Tables

**Table 1:** Primary metabolites up-regulated in mango callus infected by *Agrobacterium*.

Index	Compounds	Fold (I45/I0)	VIP Value
Gallic acid	Organic acid	15.2	1.96
Phosphoenolpyruvic acid	Organic acid	3.9	1.22
D-erythrose-4-phosphate	Phosphorylated derivatives of erythritol	4.1	1.36
Ubiquinone	Liposoluble quinone compound	11.5	1.25
Dihydrotricetin	Flavonoids	3.5	1.26
Dihydrokaempferol	Flavonoid	3.8	1.19
Cyanidin	Antioxidant	3.7	1.28

Note: I45 and I0 represent the compound content in calluses infected by *Agrobacterium* for 45 h and calluses uninfected, respectively.

**Table 2:** The top 20 genes up-regulated in callus infected by *Agrobacterium* for 45 h.

Query	Symbol	Fold (I45/I0)	Gene Annotation	Subject
LOC123211694	*MiDAHPS1*	5.2	*Mangifera indica* 3-oxoacyl-[acyl-carrier-protein] synthase	XM_044646917.1
LOC123210632	*MiDHQD1*	3	*Mangifera indica* bifunctional 3-dehydrogenate dehydratase/shikimate dehydrogenase	XM_044644036.1
LOC123236405	*MiDHQD2*	3.7	*Mangifera indica* bifunctional 3-dehydrogenate dehydratase/shikimate dehydrogenase	XM_044644035.1
LOC123207068	*MiSHD1*	2.4	*Mangifera indica* bifunctional 3-dehydrogenate dehydratase/shikimate dehydrogenase	XM_044644037.1
LOC123215035	*MiSHD2*	2.2	*Mangifera indica* bifunctional 3-dehydrogenate dehydratase/shikimate dehydrogenase	XM_044644039.1
LOC123225839	*MiCAT1*	2.9	*Mangifera indica* catalase isozyme 1	XM_044655447.1
LOC123228490	*MiCAT2*	2.6	*Mangifera indica* catalase isozyme 1	XM_044655447.1
LOC123217301	*MiGOX1*	1.7	*Mangifera indica* peroxisomal (S)-2-hydroxy-acid oxidase-like	XM_044639087.1
LOC123227499	*MiGOX2*	1.9	*Mangifera indica* peroxisomal (S)-2-hydroxy-acid oxidase-like	XM_044618393.1
LOC123204838	*MiFOX1*	3.4	*Mangifera indica* FAD-linked sulfhydryl oxidase ERV1-like	XM_044652170.1
LOC123227490	*MiFOX2*	3.8	*Mangifera indica* FAD-linked sulfhydryl oxidase ERV1-like	XM_044625447.1
LOC123206396	*MiPOD1*	2.8	*Mangifera indica* peroxidase A2-like	XM_044652117.1
LOC123208403	*MiPOD2*	2.5	*Mangifera indica* lignin-forming anionic peroxidase-like	XM_044627935.1
LOC123217403	*MiNADO1*	3.2	*Mangifera indica* respiratory burst oxidase homolog protein D-like	XM_044606776.1
LOC123228596	*MiNADO2*	3.6	*Mangifera indica* respiratory burst oxidase homolog protein A-like	XM_044630849.1
LOC123217494	*MiFLS2-1*	2.1	*Mangifera indica* LRR receptor-like serine/threonine-protein kinase FLS2	XM_044604788.1
LOC123227485	*MiMPK3-1*	1.7	*Mangifera indica* mitogen-activated protein kinase kinase 3-like	XM_044610886.1
LOC123210475	*MiMPK6-1*	2.6	*Mangifera indica* mitogen-activated protein kinase kinase 6	XM_044607731.1
LOC123210574	*MiWRKY22-1*	1.5	*Mangifera indica* WRKY transcription factor 22-like	XM_044646893.1
LOC123226579	*MiNHL10-1*	3.8	*Mangifera indica* NDR1-like protein	XM_044626069.1

Note: I45 and I0 represented gene expression in callus infected by *Agrobacterium* for 45 h and in callus uninfected, respectively.

**Table 3:** Relative expressions of genes in mango callus infected by *Agrobacterium* using qRT-PCR.

Gene	0 h	15 h	30 h	45 h	60 h
*MiDAHPS1*	1.0 ± 0.1	1.5 ± 0.1	1.9 ± 0.1	5.0 ± 0.3	4.0 ± 0.2
*MiDHQD1*	0.6 ± 0.1	0.6 ± 0.1	0.8 ± 0.1	1.9 ± 0.2	1.6 ± 0.2
*MiSHD1*	1.5 ± 0.1	1.7 ± 0.1	1.9 ± 0.2	3.6 ± 0.2	2.8 ± 0.2
*MiCAT1*	0.8 ± 0.1	0.8 ± 0.1	1.2 ± 0.1	1.9 ± 0.2	1.5 ± 0.2
*MiGOX1*	2.3 ± 0.2	2.5 ± 0.2	2.8 ± 0.2	3.9 ± 0.3	3.2 ± 0.3
*MiFOX1*	1.6 ± 0.1	1.7 ± 0.1	2.1 ± 0.2	5.6 ± 0.3	5.0 ± 0.2
*MiPOD1*	3.1 ± 0.2	3.2 ± 0.2	3.5 ± 0.2	7.3 ± 0.5	6.5 ± 0.4
*MiNADO1*	1.2 ± 0.1	1.2 ± 0.1	1.8 ± 0.1	3.6 ± 0.2	3.0 ± 0.2
*MiFLS2-1*	0.8 ± 0.1	0.8 ± 0.1	1.1 ± 0.1	1.5 ± 0.1	1.2 ± 0.1
*MiPK3-1*	5.2 ± 0.3	5.5 ± 0.3	5.8 ± 0.3	8.5 ± 0.5	7.1 ± 0.4
*MiPK6-1*	3.3 ± 0.2	3.5 ± 0.2	3.6 ± 0.3	7.2 ± 0.5	5.7 ± 0.3
*MiWRKY22-1*	1.6 ± 0.1	1.9 ± 0.2	2.1 ± 0.2	3.6 ± 0.2	2.8 ± 0.2
*MiNHL10-1*	2.6 ± 0.2	2.6 ± 0.2	2.8 ± 0.2	8.7 ± 0.5	7.2 ± 0.4

Note: 0 h, 15 h, 30 h, 45 h, and 60 h represented mango callus being infected by *Agrobacterium* for 0 h, 15 h, 30 h, 45 h, and 60 h, respectively.

**Table 4:** ROS contents in callus infected by *Agrobacterium*.

Callus	Infection Time (h)	H_2_O_2_ (μmol/mg Protein)	O^2−^ (nmol/(min mg Protein))	^•^OH (nmol/(min mg Protein))
NV	0 h	3.4 ± 0.5	6.3 ± 0.6	3.0 ± 0.2
NV	15 h	5.0 ± 0.6	5.8 ± 0.5	2.6 ± 0.2
NV	30 h	7.1 ± 0.8	10.0 ± 1.0	6.1 ± 0.5
NV	45 h	13.3 ± 1.3	17.9 ± 1.5	5.3 ± 0.4
NV	60 h	1.5 ± 0.3	8.3 ± 0.7	5.2 ± 0.4
T3SS-*AvrPto*	0 h	3.6 ± 0.4	6.1 ± 0.5	2.9 ± 0.2
T3SS-*AvrPto*	15 h	3.9 ± 0.5	6.3 ± 0.6	2.7 ± 0.2
T3SS-*AvrPto*	30 h	4.5 ± 0.5	6.6 ± 0.5	3.0 ± 0.2
T3SS-*AvrPto*	45 h	4.7 ± 0.5	6.9 ± 0.6	3.5 ± 0.3
T3SS-*AvrPto*	60 h	4.4 ± 0.6	6.5 ± 0.6	3.3 ± 0.3

Note: NV represented a callus infected by *Agrobacterium* (NV). T3SS-*AvrPto* represented the callus infected by *Agrobacterium* (T3SS-*AvrPto*).

**Table 5:** Relative expressions of top up-regulated genes in calluses infected by *Agrobacterium*.

Gene	0 h (NV)	15 h (NV)	30 h (NV)	45 h (NV)	60 h (NV)	0 h (T)	15 h (T)	30 h (T)	45 h (T)	60 h (T)
*MiDAHPS1*	1.0 ± 0.1	1.5 ± 0.1	1.9 ± 0.1	5.1 ± 0.3	4.0 ± 0.2	1.1 ± 0.1	1.2 ± 0.1	1.4 ± 0.1	2.0 ± 0.2	1.5 ± 0.1
*MiDHQD1*	0.6 ± 0.1	0.6 ± 0.1	0.8 ± 0.1	1.9 ± 0.2	1.6 ± 0.2	0.5 ± 0.1	0.5 ± 0.1	0.6 ± 0.1	1.1 ± 0.2	0.8 ± 0.1
*MiSHD1*	1.5 ± 0.1	1.7 ± 0.1	1.9 ± 0.1	3.6 ± 0.2	2.8 ± 0.2	1.5 ± 0.1	1.5 ± 0.1	1.6 ± 0.1	2.1 ± 0.2	1.8 ± 0.1
*MiCAT1*	0.8 ± 0.1	0.8 ± 0.1	1.2 ± 0.1	1.9 ± 0.2	1.5 ± 0.2	0.7 ± 0.1	0.8 ± 0.1	0.9 ± 0.1	1.3 ± 0.1	1.0 ± 0.1
*MiGOX1*	2.3 ± 0.1	2.5 ± 0.1	2.8 ± 0.1	3.9 ± 0.2	3.2 ± 0.2	2.2 ± 0.1	2.2 ± 0.1	2.3 ± 0.1	2.6 ± 0.1	2.4 ± 0.1
*MiFOX1*	1.6 ± 0.1	1.7 ± 0.1	2.1 ± 0.1	5.6 ± 0.3	5.0 ± 0.2	1.5 ± 0.1	1.6 ± 0.1	1.8 ± 0.1	2.7 ± 0.2	2.3 ± 0.1
*MiPOD1*	3.1 ± 0.1	3.2 ± 0.1	3.5 ± 0.1	7.3 ± 0.3	6.5 ± 0.2	3.0 ± 0.1	3.1 ± 0.1	3.3 ± 0.1	4.6 ± 0.2	3.8 ± 0.1
*MiNADO1*	1.2 ± 0.1	1.2 ± 0.1	1.8 ± 0.1	3.6 ± 0.2	2.8 ± 0.2	1.2 ± 0.1	1.2 ± 0.1	1.4 ± 0.1	2.1 ± 0.2	1.8 ± 0.1
*MiFLS2-1*	0.8 ± 0.1	0.8 ± 0.1	1.1 ± 0.1	1.5 ± 0.1	1.2 ± 0.1	0.8 ± 0.1	0.8 ± 0.1	0.9 ± 0.1	1.1 ± 0.1	1.0 ± 0.1
*MiPK3-1*	5.2 ± 0.2	5.5 ± 0.2	5.8 ± 0.2	8.5 ± 0.3	7.1 ± 0.3	5.1 ± 0.2	5.2 ± 0.2	5.4 ± 0.2	6.6 ± 0.2	6.1 ± 0.2
*MiPK6-1*	3.3 ± 0.2	3.5 ± 0.2	3.6 ± 0.3	7.2 ± 0.5	5.7 ± 0.3	3.2 ± 0.2	3.3 ± 0.2	3.4 ± 0.2	4.9 ± 0.2	4.5 ± 0.2
*MiWRKY22-1*	1.6 ± 0.1	1.9 ± 0.1	2.1 ± 0.1	3.6 ± 0.2	2.8 ± 0.2	1.5 ± 0.1	1.6 ± 0.1	1.9 ± 0.1	2.5 ± 0.1	2.2 ± 0.1
*MiNHL10-1*	2.6 ± 0.2	2.6 ± 0.2	2.8 ± 0.2	8.5 ± 0.5	7.2 ± 0.5	2.6 ± 0.2	2.6 ± 0.2	2.7 ± 0.2	3.6 ± 0.3	3.4 ± 0.3

Note: T represented mango calluses infected by *Agrobacterium* (T3SS-*AvrPto*). NV represented the mango callus infected by *Agrobacterium* (NV).

**Table 6:** Formulations of media used for inducing mango embryogenic callus and transformation.

Medium	Formulation
M1	B5 Mac elements, MS microelements, 4% sucrose, 10% coconut water, 500 mgL^−1^ glutamine, 0.7% agar, 0.2% activated charcoal, 3.0 mgL^−1^ 2,4-D, 0.5 mgL^−1^ KT, 0.5 mgL^−1^ ZT, pH = 5.8
M2	B5 Mac elements, MS microelements, 4% sucrose, 10% coconut water, 500 mgL^−1^ glutamine, 0.7% agar, 0.2% activated charcoal, 1.0 mgL^−1^ 2,4-D, 0.5 mgL^−1^ KT, 0.5 mgL^−1^ GA3, pH = 5.8
M3	B5 Mac elements, MS microelements, 4% sucrose, 10% coconut water, 500 mgL^−1^ glutamine, 0.7% agar, 0.2% activated charcoal, 1.0 mgL^−1^ 2,4-D, 0.5 mgL^−1^ KT, 0.5 mgL^−1^ GA3, 20 μgmL^−1^ chloromycetin, 400 mgL^−1^ timentin, with a pH of 5.8

## Data Availability

Data are contained within the article.
